# Evaluation of non-adherence to anti-retroviral therapy, the associated factors and infant outcomes among HIV-positive pregnant women: a prospective cohort study in Lesotho

**DOI:** 10.11604/pamj.2018.30.239.14532

**Published:** 2018-07-31

**Authors:** Ngomba Kadima, Tejan Baldeh, Kyaw Thin, Lehana Thabane, Lawrence Mbuagbaw

**Affiliations:** 1 Queen Mamahato Memorial Hospital, Maseru, Lesotho-CTN International Postdoctoral Fellow; 2 Department of Health Research Methods, Evidence and Impact McMaster University, Hamilton, Canada; 3 Research Coordination Unit, Room Number 326, Ministry of Health of Lesotho, Maseru, Lesotho; 4 Biostatistics Unit, Father Sean O’Sullivan Research Centre, St Joseph’s Healthcare Hamilton, Hamilton, Ontario, Canada

**Keywords:** Adherence, transmission, antiretroviral therapy, prevention of mother-to-child transmission

## Abstract

**Introduction:**

Success in addressing prevention of mother-to-child transmission of HIV depends largely on good adherence to anti-retroviral therapy (ART) by pregnant women. Knowledge of the levels of ART adherence among pregnant women is essential to inform strategies to prevent or reduce HIV transmission rates, particularly in African settings. Aim: the primary objective of this study was to measure adherence to anti-retroviral therapy (ART) among pregnant women living with human immunodeficiency virus (HIV). The secondary objectives were to determine: i) the rate of new infections among children at Mabote Filter Clinic in Maseru, Lesotho whose mothers were enrolled in PMTCT, and ii) the factors associated with non-adherence to ART among pregnant women.

**Methods:**

In this prospective cohort study, HIV-positive pregnant women receiving antiretroviral therapy (ART) for prevention of mother to child transmission (PMTCT) were followed up to delivery and their children were tested for HIV. We collected socio-demographic information, knowledge of PMTCT and adherence to ART (three-day recall and pill count) including reasons for non-adherence. We also used logistic regression to explore factors associated with non-adherence.

**Results:**

One hundred and seven women were included. The mean (standard deviation) age of the participants was 28.2 (5.7) years. Most, 81.3% (87/107), were married, only 9.3% (10/107) had a postsecondary education. Two-thirds (63.6%: 68/107) of the participants started ART because of PMTCT. Only 78.5% (84/107) of the participants had adequate knowledge of the importance of PMTCT. The three-day self-reported non-adherence rate at the first visit was 7.5% (95% confidence interval (CI): 3.7, 13.1), but up to 43.4% (95% CI: 35.2, 51.9) using pill count. The most frequently reported reasons for not adhering were: running out of pills (7.5%), nausea (5.6%) and to avoid side-effects (3.7%). Women who were employed (odds ratio (OR) 4.35; 95% CI: 1.38,14.29; p = 0.012) and at a higher gestational age (OR = 1.43; 95% CI: 1.11, 1.85; p = 0.006) were more likely to be non-adherent. Only 1 of the 77 exposed infants was found to be positive for HIV at 6 weeks after birth.

**Conclusion:**

We found a higher non-adherence rate for participants with pill count compared to a three-day adherence self-report. However, mother to child HIV transmission was relatively low. Lack of employment and relatively high gestational age were found to be predictive factors of non-adherence.

## Introduction

Lesotho has the second highest prevalence of Human Immunodeficiency Virus (HIV) in the world (25% among adults). Among the people living with HIV in Lesotho, women are the most affected and represent 60% of all HIV positive people in all age groups. The prevalence of HIV among pregnant women is estimated at 25.9% [1]. The Mother to Child Transmission (MTCT) rate in the absence of prevention of mother to child transmission (PMTCT) interventions ranges from 20-45%, but can be reduced to less than 5% with effective adherence to antiretroviral therapy (ART). In Lesotho, 60% of all women aged 15+ are HIV positive receiving ART [1]. About 13,000 children are living with HIV in Lesotho [1]. In 2013, Lesotho adopted The WHO guidelines recommending initiation of combination antiretroviral therapy for all HIV-infected pregnant or breast-feeding women, regardless of their CD4 cell count as soon as pregnancy is discovered (option B+) [2]. PMTCT has contributed to reducing the number of new infections in children, with a drop from 4400 new infections in 2009 to 1300 in 2015 [3]. However, non-adherence to ART may compromise the effectiveness of PMTCT. Given the high incidence of HIV in Lesotho and among children in particular, this study sought to evaluate adherence to antiretroviral therapy (ART) among pregnant women living with HIV in Lesotho. This study also aimed to determine: i) the rate of new infections among children at Mabote Filter Clinic in Maseru, Lesotho whose mothers were enrolled in PMTCT and ii) the factors associated with non-adherence to ART among pregnant women.

## Methods

We conducted a prospective cohort study at the Mabote Filter clinic at Maseru in Lesotho. 107 HIV positive pregnant women were followed until their child was postnatally tested for HIV. Pregnant women living with HIV were invited to participate in the study. After consent was obtained, participants were individually brought into a separate room and interviewed for 10-15 minutes by counselors. Between March 23, 2016 and June 25, 2016, 107 participants were interviewed. Mabote Filter Clinic was chosen for the study because of its convenience and accessibility to researchers. Mabote Filter clinic is located in Ha Mabote a village approximately 12 km from the city center. The clinic caters to the people living in Mabote and the surrounding villages estimated to be about 60,000 people. Mabote Filter Clinic provides the following services: HIV testing and counselling, HIV care and treatment, ANC clinic, maternity, family planning, screening of cervical cancer, and under-five primary care. All confirmed HIV positive pregnant women were eligible. HIV tests were conducted using Determine and Unigold. Pregnancy was confirmed with urine Beta human chorionic gonadotrophin (HCG0. Consent to participate was required prior to enrollment. Patients were excluded from participating in this study if they did not live in Lesotho at the time of the study. Socio-demographic data were collected, in addition to perceived predictors of adherence and their relative importance, and subject's level of knowledge on the importance of adherence to ART. Each participant was booked for 4 antenatal care (ANC) visits. At each visit, adherence levels were evaluated by self-report and pill count. Participants were encouraged to bring back the remaining drugs for pill count at each visit. For self-reported adherence, the participants were asked whether they had missed any medication doses during the three days preceding the interview.

A categorical variable was constructed reflecting two levels of adherence during a three days period namely “missed” (non-adherent, <95%) and “not missed” (adherent, ≥95%) taking medication doses during the past three days. Pill-count adherence was calculated by counting the remaining doses of medication and assuming that the remaining pills, in excess of the expected number, represented missed doses. The percentage of adherence was calculated by subtracting the number of remaining pills from the number of dispensed pills, divided by the expected number of pills to be taken and the result multiplied by 100. Adherence by pill count at the first visit was used as our primary measure. The survey was conducted in either English or Sesotho in a private setting, by one of the patient-counseling officers of the clinic, the principal investigator of this study, and a locally trained data collector. The data collector was trained to assist with administration of the questionnaires. All HIV-infected pregnant women enrolled in the study post-delivery were contacted by telephone and given a 2 weeks post-partum follow-up date for their infants. During the follow-up visit emphasis was placed on giving daily nevirapine to the infant. At 6 weeks blood was collected from all infants so that transmission of HIV could be determined via HIV deoxy ribonucleic acid (DNA) polymerase chain reaction (PCR) test. Data was entered into an electronic data base and analyzed with IBM SPSS Statistics for Windows, Version 22.0. The proportion of HIV positive pregnant women who did not adhere to anti-retroviral therapy was determined. Descriptive statistics were reported as counts and percentages or mean and standard deviation. The factors associated with adherence were investigated in univariate and multivariable models. Logistic regression was used to determine the odds of being non-adherent. The following factors were inserted as covariates in a block: age (years), level of education (none, primary, secondary and above), occupation (employed, unemployed), knowledge of PMTCT (adequate versus not adequate) and the reason why the woman was on ART (PMTCT or other). Goodness of fit was assessed using Hosmer and Lemeshow's test. Statistical significance was set at the level of 0.05. Odds ratios (OR) and 95% confidence intervals (CI) are reported. The rate of new transmissions was defined as the number of children with a positive HIV DNA PCR test at six weeks divided by the number of live births. The rate was reported with 95% confidence intervals.

## Results

**Figure 1:**
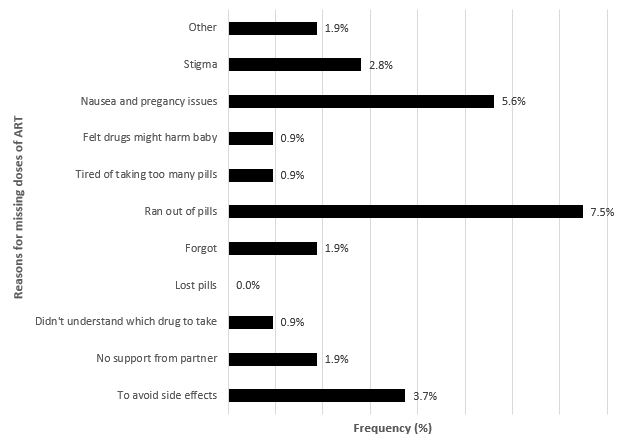
Frequency of reasons for missing doses of ART (n = 105)

The mean age (SD) of participants was 28.2 (5.7) years. Almost all the participants (87; 81.3%) of participants were married or living with a partner. Only 10 (9.3%) of the respondents had post-secondary education. A full description of participants' baseline characteristics by level of adherence (pill count at first visit) is reported in Table 1. Relatively few, 7.5% (n = 8/107), had non-adherence in the three days prior to the interview at the first visit. 43.4% (46/106) of the participants had non-adherence (pill count), with mean (SD) of 89.2% (15.7%). At the second visit, 5.6% (n = 6/77) of the participants had non-adherence (pill count) with a mean (SD) of 98.1% (6.4%). The most frequent reasons for not adhering to ART were running out of pills (7.5%) are outlined in Figure 1. After adjusting for potential confounders, women who were employed (OR 4.35; 95% CI: 1.38,14.29; p = 0.012) and at a higher gestational age (OR = 1.43; 95% CI: 1.11, 1.85; p = 0.006) more likely to be non-adherent. The Hosmer and Lemeshow Test for model fit indicated good fit (p = 0.148). The complete univariate and multivariable analyses for the non-adherence of pregnant women to ART are reported in Table 2. Seventy-seven HIV exposed infants were born to study participants during the study period of which only 1 (1.3%, 95%CI: 0.3% - 0.7%) was found positive for HIV-DNA PCR at the age of 6 weeks. The woman who was involved in the only case of transmission had suboptimal pill count adherence at first visit but optimal pill count adherence at the second visit.

**Table 1:** Baseline characteristics by level of adherence (pill count) at first interview

Variable	Adherent n (%) 60 (56.1)	Non-Adherent n (%) 46 (43.4)	Total n (%) 107 (100)
Age (years): mean (SD)	27.72 (5.42)	28.91 (6.12)	28.23 (5.70)
Gestational age (months): mean (SD)	5.38 (2.11)	6.50 (1.56)	5.76 (2.0)
**Level of education: n (%)**			
Primary	19.0 (31.7)	14.0 (30.4)	33.0 (30.8)
Secondary	33.0 (55.0)	30.0 (65.2)	64.0 (59.8)
Post-Secondary	8.0 (13.3)	2.0 (4.3)	10.0 (9.3)
**Marital status: n (%)**			
Unmarried	10.0 (16.7)	10.0 (21.7)	20.0 (18.7)
Married on living together	50.0 (83.3)	36.0 (78.3)	87.0 (81.3)
**Occupation: n (%)**			
Unemployed	50.0 (83.3)	29.0 (63.0)	79.0 (73.8)
Employed	10.0 (16.7)	17.0 (37.0)	28.0 (26.2)
**Knowledge on PMTCT: n (%)**			
Inadequate	16.0 (26.7)	7.0 (15.2)	23.0 (21.5)
Adequate	44.0 (73.3)	39.0 (84.8)	84.0 (78.5)
**Reason for ART: n (%)**			
PMTCT	42.0 (70.0)	25.0 (54.3)	68.0 (63.6)
Other	18.0 (30.0)	21.0 (45.7)	39.0 (36.4)

**Table 2:** Univariate and multivariable analyses for the non-adherence to ART

Variables	Crude OR (95% CI)	Adjusted OR (95% CI)	P-value
**Age (years)**	1.04 (0.97 to 1.11)	0.97 (0.89 to 1.06)	0.578
**Stage of Pregnancy (months)**	1.39 (0.52 to 1.72)	01.43 (1.11 to 1.85)	0.006
**Level of Education**			
Primary	1	1	0.261
Secondary	2.94 (0.54 to 16.67)	1.27 (0.48 to 3.45)	0.637
Post-secondary	3.57 (0.71 to 20.0)	0.29 (0.05 to 1.85)	0.191
**Marital status**			
Unmarried	1	1	---
Married or living			
together	1.39 (0.52 to 3.70)	1.18 (0.38 to 3.70)	0.779
**Occupation**			
Unemployed	1	1	---
Employed	2.94 (1.19 to 7.14)	4.34 (1.39 to 14.29)	0.012
**PMTCT knowledge**			
Inadequate	1	1	---
Adequate	0.49 (0.18 to 1.32)	1.37 (0.42 to 4.35)	0.605
**Reason on ART**			
Other	1	1	---
PMTCT	1.96 (1.14 to 4.34)	0.45 (0.18 to 1.15)	0.095

## Discussion

We noted a discrepancy between pill counts and self-reports, progressive decreases in non-adherence over time and a successful PMTCT program. Self-reported adherence is relatively easy to measure and easy to interpret. However, it is prone to social desirability bias and recall bias. Even though we chose a shorter period of recall (three days), self-reported adherence rates did not reflect pill counts. Even though pill counts may also have errors, this discrepancy warrants concern and points to the need for simple accurate measures of adherence in a clinical setting. Non-adherence at the first visit was high, despite adequate knowledge of the importance of PMTCT. This is in contrast to another study in Botswana in which the level of knowledge was directly proportional to the level of adherence among PMTCT clients [4]. The increments in adherence may point to the effect of repeated adherence counselling and would be suggestive that multiple sessions may create a sustained effect. Furthermore, the second visit pill count SD was higher than first visit, suggesting that the women participating in the study were adjusting to PMTCT, or those who were not adhering did not attend their second visit to the clinic and/or withdrew from the study. The adherence rates presented in this study are higher than in other parts of Africa among pregnant women (54%) [5] and non-pregnant women (~ 21%) [6, 7]. This may be because pregnant women on PMTCT are highly motivated to adhere to their medication, being keen to prevent vertical transmission to their children. Also, pregnant women on PMTCT typically visit the clinic on a monthly basis for antenatal services and collection of their antiretroviral medication. This gives the healthcare workers a good opportunity to carry out continuous adherence counseling to ensure high adherence to therapy. The cited reasons for non-adherence among the participants who did not demonstrate optimum adherence to treatment were: running out of pills before the required time and nausea. However, in the regression model, only employment and gestational age were found to be associated with non-adherence. In other studies, issues such as cost, stigma, travel/migration and side effects have been reported as barriers to adherence in non-pregnant adult patients [6]. Antiretroviral drugs are presently given free of charge to citizens of Lesotho, so the cost of medication did not play any role in the observed levels of adherence in this study. Issues such as barriers to enrollment, lack of male partner support, stigma and disclosure [8] were not found in this study. The higher levels of non-adherence in women who were employed might indicate difficulties in taking medication while at work. This may be due to concerns about stigma. It is unclear why non-adherence was higher among women at a higher gestational age but could be the effect of adherence waning over time. The rate of HIV transmission in this study was relatively low compared to national averages. This data suggests that Option B+ confers some amount of protection in the absence of optimal adherence.

## Conclusion

Adherence to ART is critical for the success of PMTCT programs. Pregnant women living with HIV in Lesotho are likely to exhibit non-adherence to HIV therapy. Women in Lesotho are likely to under-report their non-adherence to HIV therapy via self-report compared to pill count. Pregnant women living with HIV face challenges in adhering to medication, but can benefit from continuous adherence counselling. Sociodemographic characteristics, such as gestational age and employment status, increase the levels of non-adherence in women. Transmission of HIV from these women to their children is relatively low. Implementing Option B+ coupled to continuous adherence counselling would likely reduce the rates of transmission even further.

### What is known about this topic

- The World Health Organization recommends that all pregnant women with HIV be started on antiretroviral therapy (Option B+);
- Many countries have adopted Option B+ and are progressively rolling it out.

### What this study adds

- Option B+ can be implemented effectively in a low-resource setting like Lesotho;
- Adherence to ART in PMTCT programs can be enhanced by continuous counselling and increases over time;
- Sociodemographic characteristics such as gestational age and employment status increase the levels of non-adherence in women.

## Competing interests

The authors declare no competing interests.
